# SARS-CoV-2 N protein coordinates viral particle assembly through multiple domains

**DOI:** 10.1128/jvi.01036-24

**Published:** 2024-10-16

**Authors:** Yuewen Han, Haiwu Zhou, Cong Liu, Weiwei Wang, Yali Qin, Mingzhou Chen

**Affiliations:** 1 State Key Laboratory of Virology and Modern Virology Research Center, College of Life Sciences, Wuhan University, Wuhan, China; 2 School of Life Sciences, Hubei University, Wuhan, China; 3 Hubei Jiangxia Laboratory, Wuhan, China; St. Jude Children's Research Hospital, Memphis, Tennessee, USA

**Keywords:** SARS-CoV-2, nucleocapsid protein, membrane protein, genomic RNA, assembly, replication

## Abstract

Increasing evidence suggests that mutations in the nucleocapsid (N) protein of severe acute respiratory syndrome coronavirus 2 (SARS-CoV-2) may enhance viral replication by modulating the assembly process. However, the mechanisms governing the selective packaging of viral genomic RNA by the N protein, along with the assembly and budding processes, remain poorly understood. Utilizing a virus-like particles (VLPs) system, we have identified that the C-terminal domain (CTD) of the N protein is essential for its interaction with the membrane (M) protein during budding, crucial for binding and packaging genomic RNA. Notably, the isolated CTD lacks M protein interaction capacity and budding ability. Yet, upon fusion with the N-terminal domain (NTD) or the linker region (LKR), the resulting NTD/CTD and LKR/CTD acquire RNA-dependent interactions with the M protein and acquire budding capabilities. Furthermore, the presence of the C-tail is vital for efficient genomic RNA encapsidation by the N protein, possibly regulated by interactions with the M protein. Remarkably, the NTD of the N protein appears dispensable for virus particle assembly, offering the virus adaptive advantages. The emergence of N* (NΔN209) in the SARS-CoV-2 B.1.1 lineage corroborates our findings and hints at the potential evolution of a more streamlined N protein by the SARS-CoV-2 virus to facilitate the assembly process. Comparable observations have been noted with the N proteins of SARS-CoV and HCoV-OC43 viruses. In essence, these findings propose that β-coronaviruses may augment their replication by fine-tuning the assembly process.

**IMPORTANCE:**

As a highly transmissible zoonotic virus, severe acute respiratory syndrome coronavirus 2 (SARS-CoV-2) continues to evolve. Adaptive mutations in the nucleocapsid (N) protein highlight the critical role of N protein-based assembly in the virus’s replication and evolutionary dynamics. However, the precise molecular mechanisms of N protein-mediated viral assembly remain inadequately understood. Our study elucidates the intricate interactions between the N protein, membrane (M) protein, and genomic RNA, revealing a C-terminal domain (CTD)-based assembly mechanism common among β-coronaviruses. The appearance of the N* variant within the SARS-CoV-2 B.1.1 lineage supports our conclusion that the N-terminal domain (NTD) of the N protein is not essential for viral assembly. This work not only enhances our understanding of coronavirus assembly mechanisms but also provides new insights for developing antiviral drugs targeting these conserved processes.

## INTRODUCTION

Since its emergence in late 2019, severe acute respiratory syndrome coronavirus 2 (SARS-CoV-2) has spread extensively among humans, leading to a global health crisis and demonstrating a remarkable ability for adaptive evolution (1–4). Mutations in the nucleocapsid (N) protein, in addition to the spike protein, have been identified in numerous prevalent strains, playing a crucial role in SARS-CoV-2’s continuous adaptation to human hosts (5–8). These mutations in the N protein contribute to enhanced viral replication and pathogenicity by modulating the assembly process (9–12). This highlights the significance of comprehending the assembly process mediated by the N protein to better understand the virus’s evolution and transmission dynamics.

SARS-CoV-2, like other coronaviruses, possesses a 30 kb genome that undergoes discontinuous transcription to generate sgRNA encoding various structural and accessory proteins (13, 14). Among these proteins, the N protein plays a critical role in vital processes such as replication, transcription, genome packaging, and virion assembly (15–17). During the assembly phase, the N protein is particularly important for facilitating the encapsidation of genomic RNA, a process whose understanding has been significantly enhanced by elucidating its structure. The N protein consists of modules comprising intrinsically disordered regions (IDRs) and conserved domains, namely the N-terminal domain (NTD) and the C-terminal domain (CTD), linked by a flexible linker region (LKR) (18, 19). The N-arm, LKR, and C-tail constitute the IDRs, while the NTD and CTD are bordered by the N-arm and C-tail (18). Notably, the C-terminal IDR of the N protein plays a critical role in viral assembly through its interaction with the M protein (20–22). Within the N-NTD of SARS-CoV-2, the β2′–β3′ β-hairpin forms a positively charged pocket at its junction with the core structure, suggesting a potential RNA binding site (19). Additional RNA binding capability is found in the positively charged groove on the helical face of the N-CTD dimer, specifically formed by residues K256, K257, K261, and R262 (18). Furthermore, the N-terminal IDR and the LKR exhibit RNA-binding capabilities, contributing to enhanced binding affinity and allosteric enhancements (23). This synergistic binding mechanism enables the N protein to interact with RNA cooperatively, indicating that the NTD, CTD, and certain disordered regions collectively contribute to ribonucleoprotein (RNP) packaging.

Coronaviruses rely on the M protein for driving particle morphogenesis (24, 25). Upon synthesis within host cells, the M protein localizes to the endoplasmic reticulum-Golgi intermediate compartment (ERGIC) and acts as a scaffold for recruiting other viral structural proteins (26). Cryo-electron microscopy studies have revealed that the M protein forms a dimer with a mushroom-like shape, comprising two transmembrane domain-swapped three-helix bundles and two intravirion domains (24, 25). Additionally, the M protein can assemble into higher-order oligomers. The dimerization of the M protein is facilitated by both its transmembrane helices and the C-terminal β-sheet sandwich domain. Furthermore, M protein oligomerization, coupled with associated membrane curvature, can influence the formation of spherical virion particles. The intravirion domain of the M protein is crucial for virion assembly and release (27). Post-translational modifications, such as N-terminal glycosylation and ubiquitination, may play significant roles in regulating M protein assembly and budding (27–29).

The assembly of coronaviruses is intricately orchestrated by the interplay between the N protein and M protein. Current understanding posits that the M protein of SARS-CoV-2 plays a pivotal role in orchestrating the coordinated recruitment of the N protein and RNA, primarily through its positively charged intravirion domain (24). Recent studies suggest that the N protein wraps around the viral genome and drives subsequent assembly processes via phase separation mechanisms (22, 30–34). Various research teams have demonstrated the ability of the N protein to undergo phase separation *in vitro* with different RNA species, a behavior regulated by factors such as pH, salt concentration, and RNA abundance (31, 34, 35). This phase separation process is intricately regulated by viral RNA sequences and structures, dictating the condensation and dissolution of N protein-RNA condensates (35). Interestingly, it has been observed that the N protein can undergo phase separation not only with genomic RNA but also with the M protein, implying a potential role for membrane-associated M proteins in recruiting N+RNA condensates during virion formation (22). Furthermore, phase separation driven by RNA and M protein interactions involves distinct domains of the N protein. While the essential role of the N protein in virus assembly is well-established, the precise mechanisms governing its selective packaging of viral genomic RNA and its interaction with the M protein for virus budding remain to be fully elucidated. These ongoing investigations hold promise for a deeper understanding of coronavirus assembly mechanisms and may unveil novel targets for therapeutic interventions.

Research has observed an increase in the prevalence of concurrent mutations R203K/G204R in the N protein of SARS-CoV-2 among newly identified variants of concern or interest (9, 11, 12, 36–39). Competitive assays suggest that the 203K/204R variants exhibit superior replication compared to the original R203/G204 variants, likely due to enhancements in RNP assembly (11). Mourier et al. also verified that these mutations significantly increase binding affinity to viral RNA (9). Syed et al. used SARS-CoV-2 virus-like particles (VLPs) to illustrate that N mutations and particle assembly mechanisms could elucidate the increased spread of variants, including B.1.617.2 (Delta, containing the R203M mutation) (10). Moreover, Thorne et al. observed the emergence of N* (NΔN209) mutants across multiple viral strains, characterized by a deletion of 209 amino acids from the N-terminus (40). Subsequently, Adly et al. demonstrated that the N* variant maintains an RNP conformation comparable to that of the wild-type (WT) N protein (41). Although it remains uncertain whether the individual N* variant can assemble into an infectious viral particle, the emergence of these mutants signals the potential for coronaviruses to employ modulation of the viral assembly process as a novel evolutionary strategy.

In this study, we have unraveled an assembly mechanism centered around the CTD of the N protein, a feature commonly observed in β-coronaviruses. The isolated CTD of the N protein lacks the capability to initiate assembly budding; it relies on assistance from the NTD or the LKR to establish RNA-dependent interactions with the M protein and enable budding. Moreover, the C-tail of the N protein engages in RNA-independent interactions with the M protein, although these interactions alone are insufficient to drive budding. Notably, the presence of the C-tail is crucial for achieving genomic RNA encapsidation. Interestingly, our findings also indicate that the NTD is dispensable for packaging genomic RNA and budding. Furthermore, we observed that the N* variant retains the ability to package viral genomic RNA and facilitate budding, providing additional support for our conclusions. Overall, our study sheds light on the complex interplay between the N protein, M protein, and genomic RNA during the particle assembly process of SARS-CoV-2.

## RESULTS

### Co-expression of the M and N proteins is sufficient to form virus-like particles

**Fig 1:**
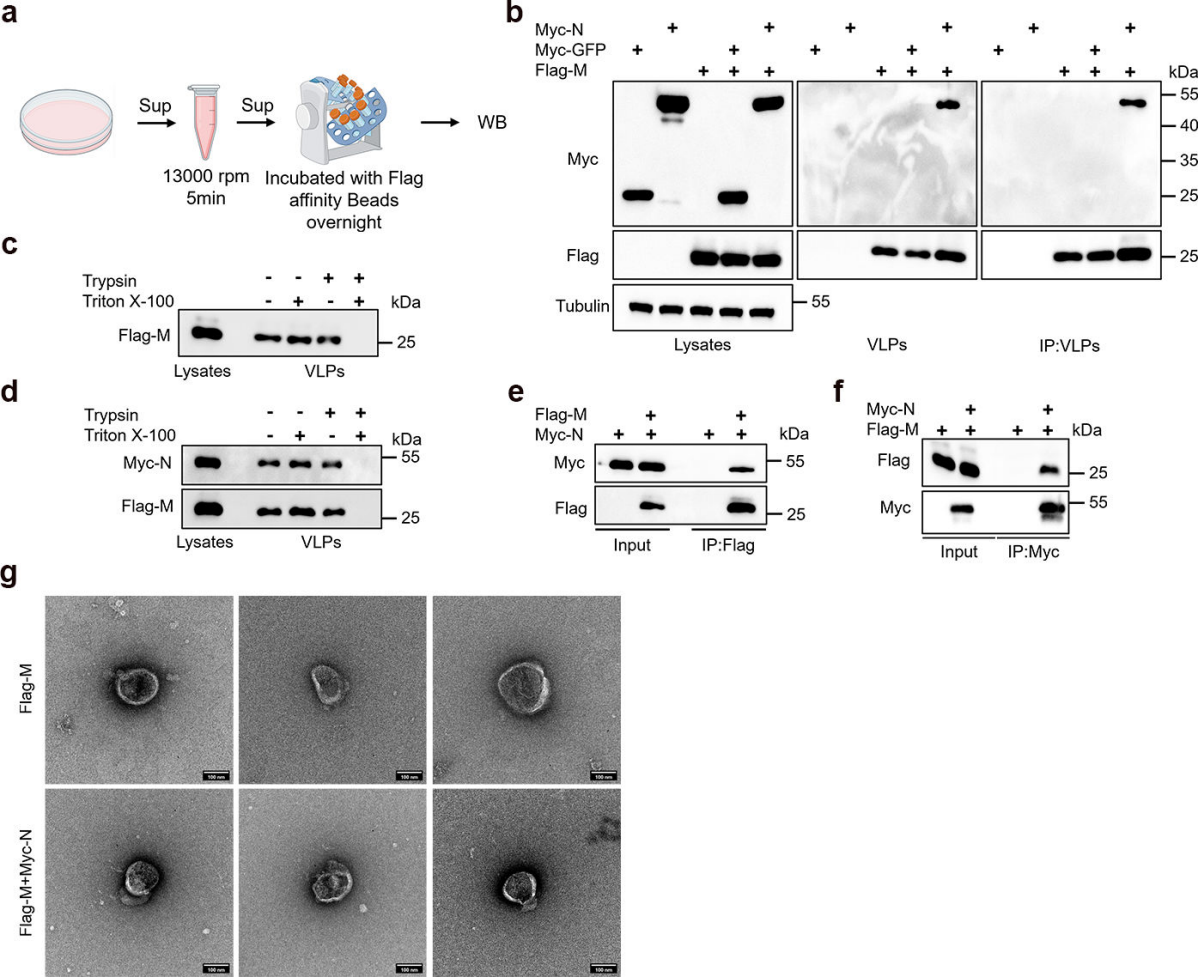
The M and N proteins of SARS-CoV-2 have the capability to assemble and release VLPs. (a) Flowchart of VLP capture using flag affinity beads. (b) The N protein can be specifically packaged into M-VLPs. Transfections of Myc-GFP, Myc-N, Flag-M, and co-transfections of Myc-GFP+Flag-M and Myc-N+Flag-M were individually conducted in HEK293T cells and incubated for 48 hours. Subsequently, half of the cell culture supernatant underwent ultracentrifugation for VLP isolation, while the remaining half was captured using Flag agarose beads, following the outlined protocol in the Materials and Methods. This was followed by western blot (WB) analysis. (c and d) Protease protection analysis of M-VLPs and M+N-VLPs. HEK293T cells were transfected with Flag-M and Flag-M+Myc-N constructs and incubated for 48 hours. A protease protection assay was then performed on the VLPs according to the outlined methodology in the Materials and Methods, followed by WB analysis. (e and f) Analysis of the interaction between M Protein and N Protein. HEK293T cells were transfected with Myc-N and Flag-M for 36 hours, followed by Flag or Myc immunoprecipitation (IP) and subsequent WB analysis. (g) Representative TEM graphs of M and M+N VLPs. VLP was prepared and then visualized by TEM. Scale bar 100 nm.

VLP systems serve as effective tools for investigating virus assembly mechanisms. Prior studies have demonstrated the ability of the M and N proteins of SARS-CoV-2 to assemble into VLPs (42, 43). Besides using ultracentrifugation to isolate VLPs, we also employed immunoprecipitation as a complementary method (Fig. 1a). The results show that both the individually expressed M protein and the co-expressed M and N proteins were detected in VLPs obtained through ultracentrifugation, whereas the control GFP, whether expressed alone or with M protein, was not released into the supernatant (Fig. 1b). Similarly, in the immunoprecipitated VLPs, we detected both the individually expressed M protein and the co-expressed M and N proteins. This further confirms that the N protein is specifically packaged into M-VLPs. Subsequently, to further confirm that M-VLPs and M+N-VLPs possess intact membrane structures, we subjected the VLPs obtained from ultracentrifugation to treatments with trypsin alone, Triton X-100 alone, and a combination of both. We observed that M protein and N protein were only completely digested when trypsin and Triton X-100 were used together (Fig. 1c and d). The fact that N protein can be packaged into VLPs by M protein suggests an interaction between them, which we further validated. The results demonstrated that immunoprecipitation of either M protein or N protein effectively co-precipitated the other protein, confirming their interaction (Fig. 1e and f). To confirm the ability of M protein alone or in combination with N protein to form VLPs, we used transmission electron microscopy (TEM) to examine their morphology. Immunoprecipitation was employed to specifically enrich VLPs, minimizing interference from other vesicular structures. TEM analysis showed that VLPs formed by M protein alone or co-expressed with N protein exhibited irregular spherical shapes, averaging around 100 nm, consistent with typical coronavirus VLP morphology (Fig. 1g). We then used this convenient assay to investigate the mechanism by which N protein regulates viral assembly.

### Mapping the critical region required for N protein budding

**Fig 2:**
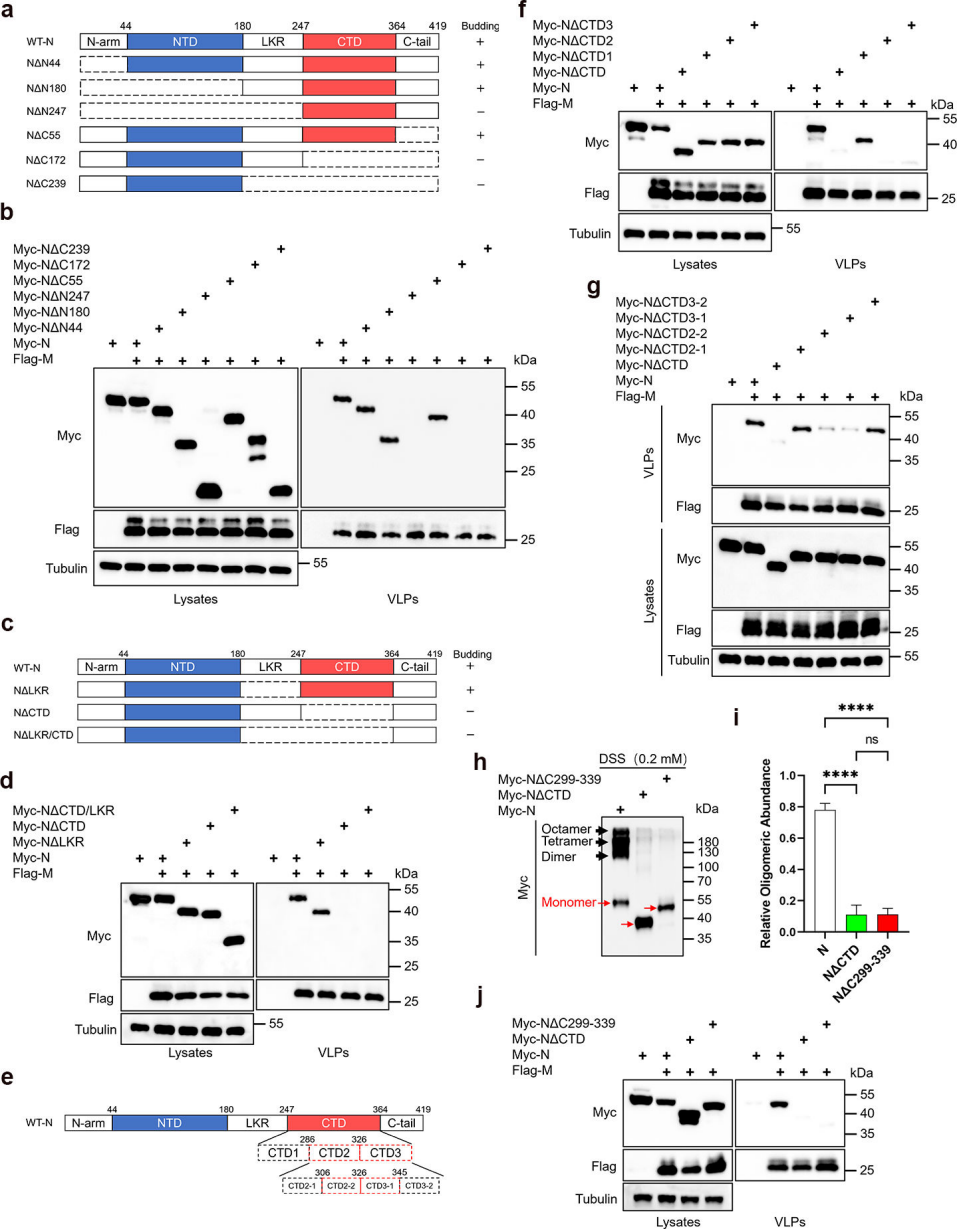
The CTD of N protein is essential for its budding. (a, c) Summary of the budding status of N protein truncations. (b, d, f, g, j) Analysis of the budding ability of N protein truncations. HEK293T cells were transfected with Myc-tagged N protein truncations along with Flag-M for 48 hours. A VLP assay was conducted via ultracentrifugation, followed by WB analysis. (e) The schematic diagram of the N protein truncations. (h and i) Analysis of oligomerization of N protein truncations. HEK293T cells were transfected with Myc-tagged N protein truncations for 36 hours, cells were cross-linked by being treated with disuccinimidyl suberate (DSS) for 30 minutes, and then lysates were analyzed via WB. Red arrows indicate monomers and black arrows indicate oligomers. The oligomeric states of the N protein and its mutants were quantified using ImageJ, with the oligomerization state indicated by the ratio of oligomer band intensity to the total band (monomers and oligomers) intensity. The oligomeric states of the N protein and its mutants were quantified using ImageJ, (*n* = 3). Data in i is represented as mean ± SD and was analyzed by one-way analysis of variance (ANOVA). *P < 0.05, **P < 0.01, ***P < 0.001, ****P < 0.0001.

To investigate the mechanism of N protein assembly and budding, we generated six truncated N protein mutants to evaluate their budding capabilities. Our findings show that deleting the ARM (NΔN44), alone or with the NTD (NΔN180), does not impact the N protein, while further removal of LKR (NΔN247) abolishes its budding capability. The deletion of the C-tail (NΔC55) does not impair the budding capability of the N protein. However, when the CTD (NΔC172) is further deleted, the N protein completely loses its budding ability. Further deletion of LKR (NΔC239) also results in a complete loss of budding capability (Fig. 2a and b). Subsequently, we generated mutants lacking only the LKR, only the CTD, and mutants lacking both the LKR and CTD to investigate their roles in budding. Our findings indicate that the absence of the CTD alone abolishes the N protein’s budding capability, while the exclusive deletion of the LKR does not impair the N protein’s ability to bud (Fig. 2c and d). These results underscore the essential nature of the CTD as the domain crucial for N protein budding.

Next, We generated two sets of deletion mutants targeting internal regions of the CTD and evaluated their budding capabilities. We observed a significant reduction in budding capability in mutants NΔCTD2-2 and NΔCTD3-1 (Fig. 2e through g). Based on previous research, we found that amino acids 299-339 of the N protein encompass the α-helix and β-sheet forming the dimerization interface (18). Consequently, we constructed a new mutant, termed NΔC299-339, to evaluate the role of this segment in N protein oligomerization and budding. We treated cells transfected with N and its mutants with disuccinimidyl suberate (DSS) and evaluated their oligomerization capacity by analyzing the proportion of oligomer bands relative to the total grayscale. Due to the blurred boundaries between different oligomer bands formed after DSS crosslinking, we measured the grayscale values of all oligomer bands. Additionally, we validated the oligomerization ability of the N protein and its mutants through co-immunoprecipitation experiments. The results indicate that deletion of this segment not only causes the N protein to lose its oligomerization ability but also almost abolishes its budding capability (Fig. 2h through j; Fig. S1a).

### Oligomerization mediated by the N protein CTD is essential for its budding

**Fig 3:**
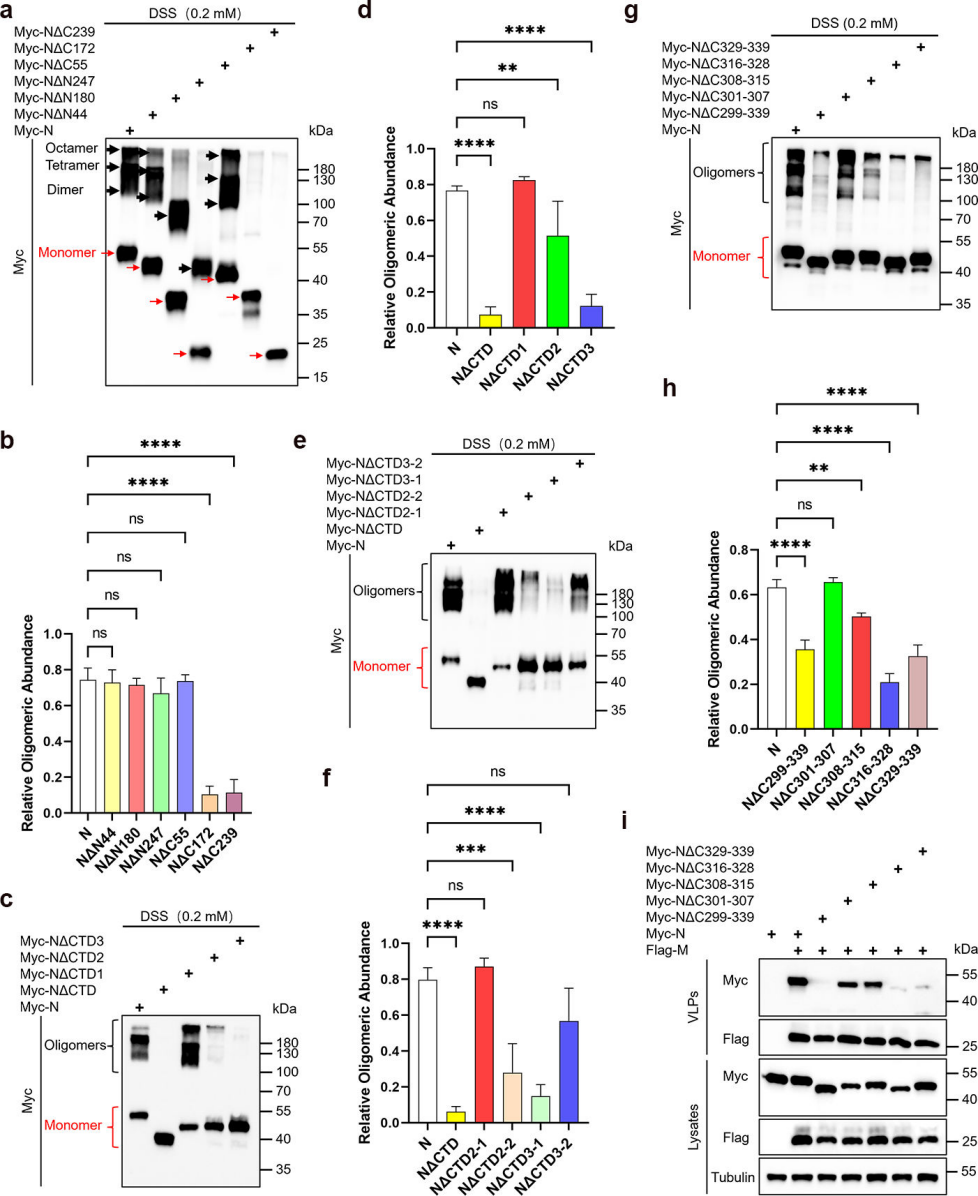
Oligomerization of the N protein is crucial for its budding. (a–h) Analysis of oligomerization of N protein truncations. HEK293T cells were transfected with Myc-tagged N protein truncations for 36 hours, cells were cross-linked by being treated with DSS for 30 minutes, and then lysates were analyzed via WB. Red arrows indicate monomers and black arrows indicate oligomers. The oligomeric states of the N protein and its mutants were quantified using ImageJ, b (*n* = 4), d (*n* = 4), f (*n* = 3), h (*n* = 3). (i) Analysis of the budding ability of N protein truncations. HEK293T cells were transfected with Myc-tagged N protein truncations along with Flag-M for 48 hours. A VLP assay was conducted via ultracentrifugation, followed by WB analysis. Data in b, d, f, h were represented as mean ± SD and were analyzed by one-way ANOVA. *P < 0.05, **P < 0.01, ***P < 0.001, ****P < 0.0001.

Given the critical role of N protein oligomerization in viral particle assembly, we further investigated whether all budding-competent N protein mutants retained oligomerization capability. The results showed that mutants retaining the CTD, including NΔN44 (NΔN-arm), NΔN180 (NΔN-arm/NTD), NΔN247 (NΔN-arm/NTD/LKR), and NΔC55 (NΔC-tail), all exhibited substantial oligomerization capacity (Fig. 3a and b), consistent with the results of the co-immunoprecipitation experiment (Fig. S1b). Although NΔN180 and NΔN247 appeared to primarily form dimers compared to full-length N protein and NΔN44, NΔN180 could still bud, suggesting that dimer formation may be sufficient to meet the minimum requirements for assembly and budding. The ability of NΔN247 to form dimers but not to bud indicates that N protein oligomerization is a necessary but not sufficient condition for budding. We subsequently investigated the impact of different regions within the CTD on its oligomerization capability. Our findings revealed that the oligomerization ability of the previously identified non-budding mutants, NΔCTD2 and NΔCTD3, was significantly reduced (Fig. 3c and d; Fig. S1c). Further validation confirmed that the non-budding mutants NΔCTD2-2 and NΔCTD3-1 also exhibited markedly weaker oligomerization compared to the wild-type N protein (Fig. 3e and f; Fig. S1d). Additionally, deleting the β1 and β2 regions within the CTD dimerization interface significantly impaired both the oligomerization and budding capabilities of the N protein (Fig. 3g through i; Fig. S1e). These findings suggest that CTD-mediated oligomerization of the N protein is a critical prerequisite for its assembly and budding.

### The CTD of the N protein serves as the structural foundation for its interaction with the M protein

**Fig 4:**
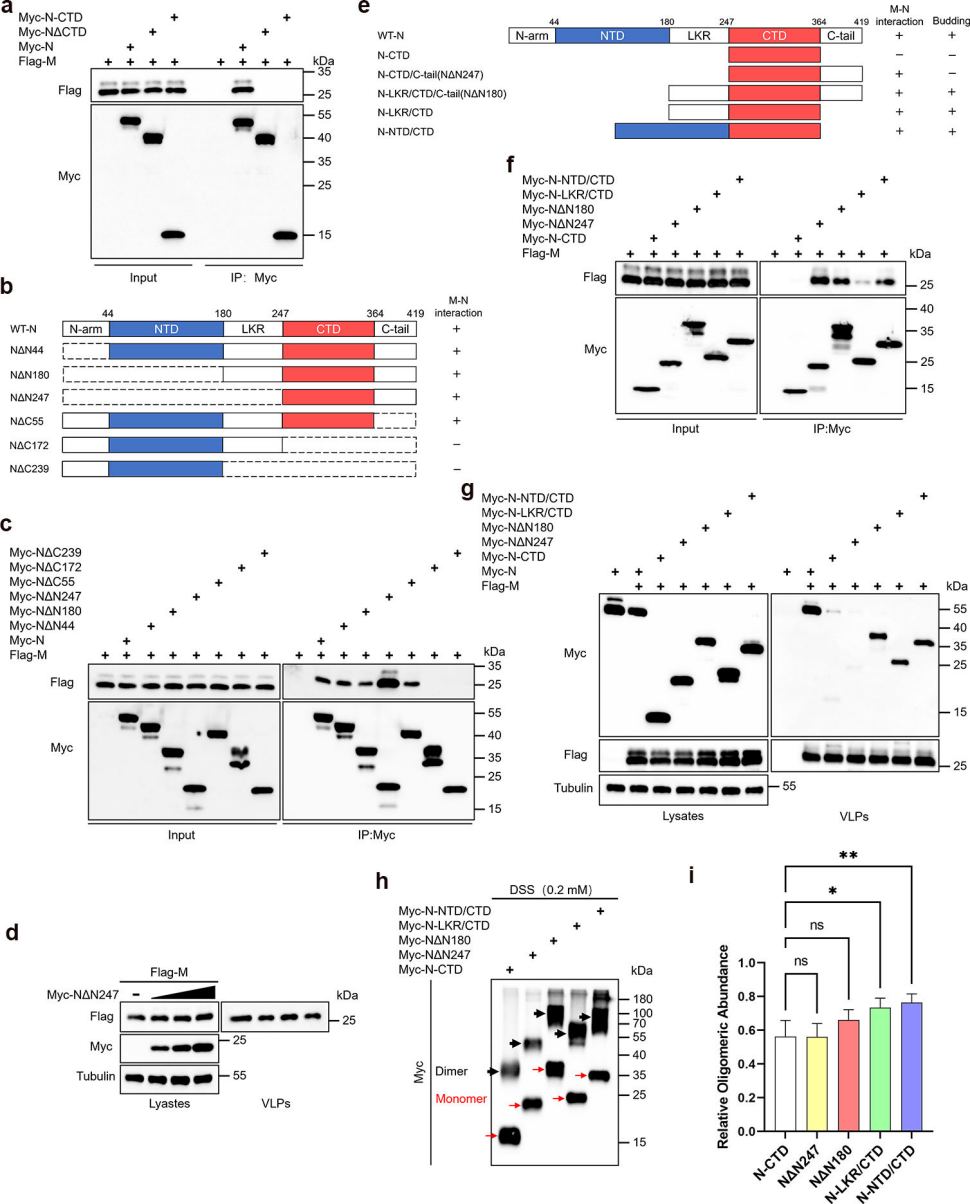
The CTD of the N protein serves as the structural basis for its interaction with the M protein. (a, c, f) Analysis of interactions between N protein truncations and M protein. HEK293T cells were transfected with Myc-tagged N protein truncations and Flag-M for 36 hours, followed by Myc IP and subsequent WB analysis. (b) Summary of the interaction status between N mutants and M protein. (d) NΔN247 does not impede M protein budding. HEK293T cells were transfected with increasing amounts of Myc-NΔN247 and Flag-M for 48 hours. VLPs budding assay was conducted via ultracentrifugation, followed by WB analysis. (e) Summary of the interaction and budding status of N-CTD related mutants with M. (g) Analysis of budding ability of N protein truncations. HEK293T cells were transfected with Myc-tagged N truncations along with Flag-M for 48 hours. A VLP assay was conducted via ultracentrifugation, followed by WB analysis. (h and i) Analysis of oligomerization of N-CTD-related mutants. HEK293T cells were transfected with N-CTD related mutants for 36 hours, cells were cross-linked by being treated with DSS for 30 minutes, and then lysates were analyzed via WB. Red arrows indicate monomers and black arrows indicate oligomers. The oligomeric states of the N protein and its mutants were quantified using ImageJ, (*n* = 4). Data in i was represented as mean ± SD and was analyzed by one-way ANOVA. *P < 0.05, **P < 0.01, ***P < 0.001, ****P < 0.0001.

The interaction between the N and M proteins is considered one of the driving forces for coronavirus assembly and budding; however, the complex relationship between them remains unclear. Firstly, we found that the deletion of CTD of the N protein disrupts its interaction with the M protein, and the isolated CTD alone does not possess the ability to interact with the M protein (Fig. 4a). Further experiments revealed that all budding-competent N protein mutants retain interaction with the M protein. (Fig. 4b and c). It is noteworthy that the non-budding NΔN247 exhibits a strong interaction with the M protein, implying that the interaction mediated by the C-tail is insufficient to package it into M-VLPs. What’s more, this interaction does not influence M protein budding (Fig. 4d). We also observed that the sole deletion of the N protein’s C-tail does not disrupt its interaction with the M protein (Fig. 4b and c). This indicates the potential involvement of other regions on the N protein in interacting with the M protein. To explore this further, we constructed two fusion mutants based on CTD, namely NTD/CTD and LKR/CTD, and analyzed their interactions with M and budding capabilities, alongside CTD and NΔN180, NΔN247 as controls. Our results reveal that, compared to CTD alone, these fusion mutants all exhibit interactions with the M protein, albeit the interaction with LKR/CTD and M protein appears weaker (Fig. 4e and f). This suggests the potential involvement of NTD and LKR in the interaction with the M protein. Moreover, the interaction mediated by NTD or LKR with the M protein is sufficient for packaging NTD/CTD and LKR/CTD into M-VLPs (Fig. 4g). Compared to the CTD, NΔN247, NΔN180, NTD/CTD, and LKR/CTD did not show significantly enhanced oligomerization ability (Fig. 4h and i; Fig. S1f). Furthermore, given that the interaction of all N protein mutants with the M protein relies on the presence of the CTD, we propose that the CTD acts as the structural basis for the N-M protein interaction.

### The role of RNA in mediating N protein and M protein interaction for N protein budding

**Fig 5:**
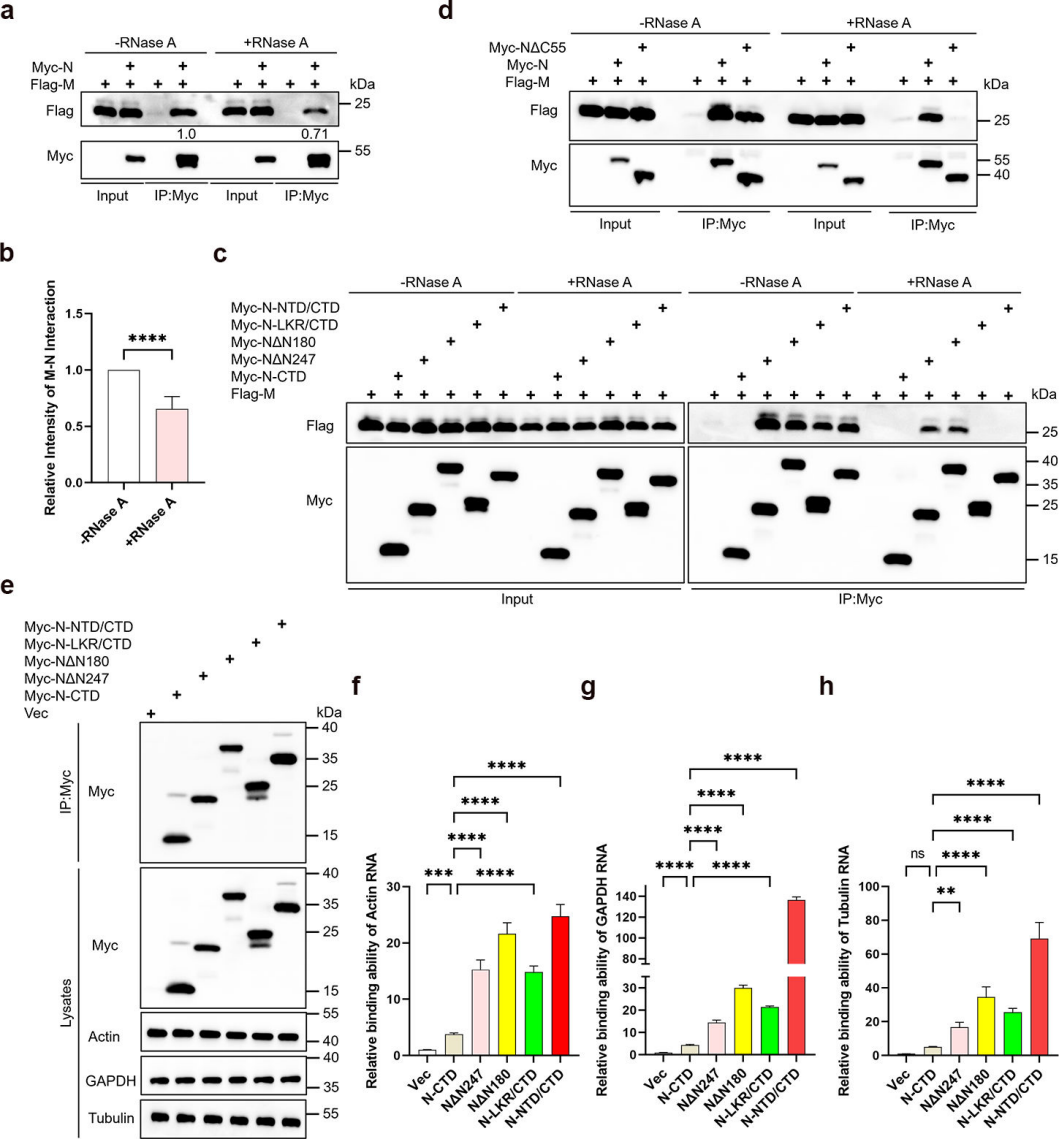
The interaction between the N protein and the M protein is regulated by RNA. (a, c, d) Analysis of RNA’s impact on the interactions between N protein truncations and M protein. HEK293T cells were transfected with Myc-N truncations and Flag-M for 36 hours. Divide the lysates into two parts: one is left untreated, and the other is treated with RNase A, followed by Myc IP and subsequent WB analysis. (b) M protein bands from co-IP experiments were quantified using ImageJ. Band intensities from RNase A-treated samples were normalized to untreated controls, and the relative values represent the strength of the M-N interaction, (*n* = 6). (e–h) Analysis of RNA binding capability of N-CTD related mutants. HEK293T cells were transfected with Myc-tagged N-CTD-related mutants for 36 hours. Cell lysates were subjected to IP with anti-Myc antibodies, followed by RNA extraction and reverse transcription quantitative PCR (RT-qPCR). The data were presented on a relative scale, with the IP Actin, GAPDH, and Tubulin RNA normalized against the cell lysate’s RNA for each sample, (*n* = 3). Both IP and cell lysate fractions were analyzed via WB. Data in b were represented as mean ± SD and were analyzed by Student’s *t*-test. Data in f–h were represented as mean ± SD and were analyzed by one-way ANOVA. *P < 0.05, **P < 0.01, ***P < 0.001, ****P < 0.0001.

The interaction between N protein and M protein, mediated by NTD or LKR, drives N protein budding. How does this interaction differ from that mediated by C-tail with M protein? Both NTD and LKR of the N protein have been reported to possess RNA-binding capabilities (44) and studies by Zhang et al. confirmed that M-N interaction is not lost in the absence of RNA (24). Thus, we speculate that RNA regulates the interaction between NTD/CTD or LKR/CTD with M. We first confirmed that RNA digestion inhibits the N-M protein interaction (Fig. 5a and b). Further analysis showed that interactions of NTD/CTD and LKR/CTD with M were abolished upon RNA digestion, while interactions of NΔN180 and NΔN247 with M were weakened but still present (Fig. 5c). This suggests that NTD or LKR interactions with M are RNA-dependent. Additionally, the interaction between NΔC55 and M was almost lost after RNA digestion (Fig. 5d). We then examined if fusing NTD or LKR with CTD increases RNA binding.

We next employed an immunoprecipitation-RT-qPCR assay using Actin, GAPDH, and Tubulin mRNA as surrogates for non-specific RNA to assess the binding affinity of different mutants. Compared to the control group, the CTD showed some RNA binding capability. Additionally, NΔN180, NΔN247, LKR/CTD, and NTD/CTD exhibited stronger binding to RNA compared to CTD alone (Fig. 5e through h). These results further suggest that RNA may play a role in modulating the interaction between these mutants and the M protein.

### The N protein is capable of selectively packaging genomic RNA

**Fig 6:**
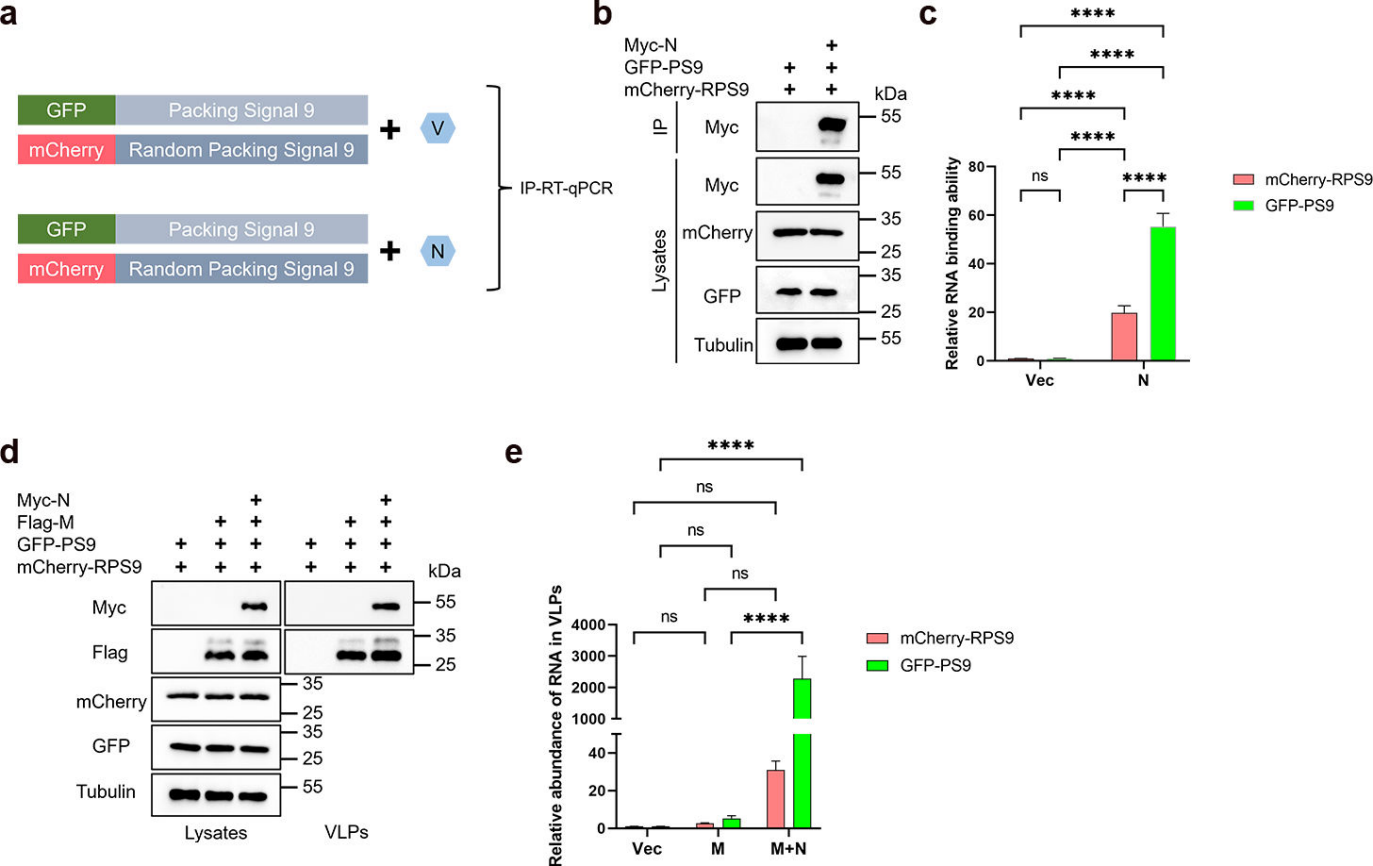
The N protein exhibits a specific affinity for encapsidating genomic RNA. (a) Schematic diagram depicting the validation of N protein’s binding affinity for genomic RNA via IP-RT-qPCR. (b and c) The N protein exhibits a higher binding affinity for genomic RNA. HEK293T cells were transfected with Myc-N along with mCherry-RPS9 and GFP-PS9 for 36 hours. Cell lysates underwent IP with anti-Myc antibodies, followed by RNA extraction and RT-qPCR. The data were presented on a relative scale, with the IP mCherry and GFP RNA normalized against the cell lysate’s mCherry and GFP RNA for each sample, (*n* = 4). Both IP and cell lysate fractions were analyzed via WB. (d and e) PS9 can be packaged into M+N-VLPs. HEK293T cells were transfected with Myc-N along with mCherry-RPS9, GFP-PS9, and Flag-M for 48 hours. Half of the cell culture supernatant was subjected to ultracentrifugation to isolate VLPs for WB analysis, while the other half was used for RNA extraction, followed by RT-qPCR. The data were presented on a relative scale, with the VLPs’ mCherry and GFP RNA normalized against the cell lysate’s mCherry and GFP RNA for each sample, (*n* = 4). Both VLPs and cell lysate fractions were analyzed via WB. Data in c and e is represented as mean ± SD and was analyzed by two-way ANOVA. *P < 0.05, **P < 0.01, ***P < 0.001, ****P < 0.0001.

Multiple studies have shown that a packaging signal exists in the β-coronavirus genome, which not only facilitates the selective packaging of genomic RNA but also plays a crucial role in viral replication and pathogenesis (10, 45, 46). Syed et al. identified PS9 at the 3' end of ORF1a/b in SARS-CoV-2 as the minimal element packaged by the N protein into virus particles, including reporter genes (10). Consequently, we utilized the PS9 sequence to investigate viral genome encapsidation mechanisms. We fused PS9 to the C-terminus of GFP and, as a control, fused a randomly scrambled version of PS9 to the C-terminus of mCherry to indicate non-specific RNA (Fig. 6a). First, we assessed the N protein’s binding affinity for different RNA types. The results showed that the N protein exhibited a significantly higher binding affinity for GFP-PS9 RNA compared to mCherry-RPS9 RNA. However, the N protein also displayed some binding to the non-specific mCherry-PS9 RNA relative to the control (Fig. 6b and c). Next, we analyzed the relative abundance of different RNAs in VLPs to determine if the N protein can package GFP-PS9 RNA. The results revealed that GFP-PS9 RNA was significantly more abundant than mCherry-PS9 RNA in M+N-VLPs. The abundance of mCherry-RPS9 RNA was similar across the control, M, and M + N VLP groups, indicating that the N protein specifically packaged GFP-PS9 RNA. Additionally, GFP-PS9 RNA was nearly absent in M-VLPs compared to the control group, demonstrating that the packaging of GFP-PS9 RNA in SARS-CoV-2 VLPs is highly dependent on the N protein (Fig. 6d and e).

### Multiple domains within the N protein orchestrate its binding to and packaging of genomic RNA

**Fig 7:**
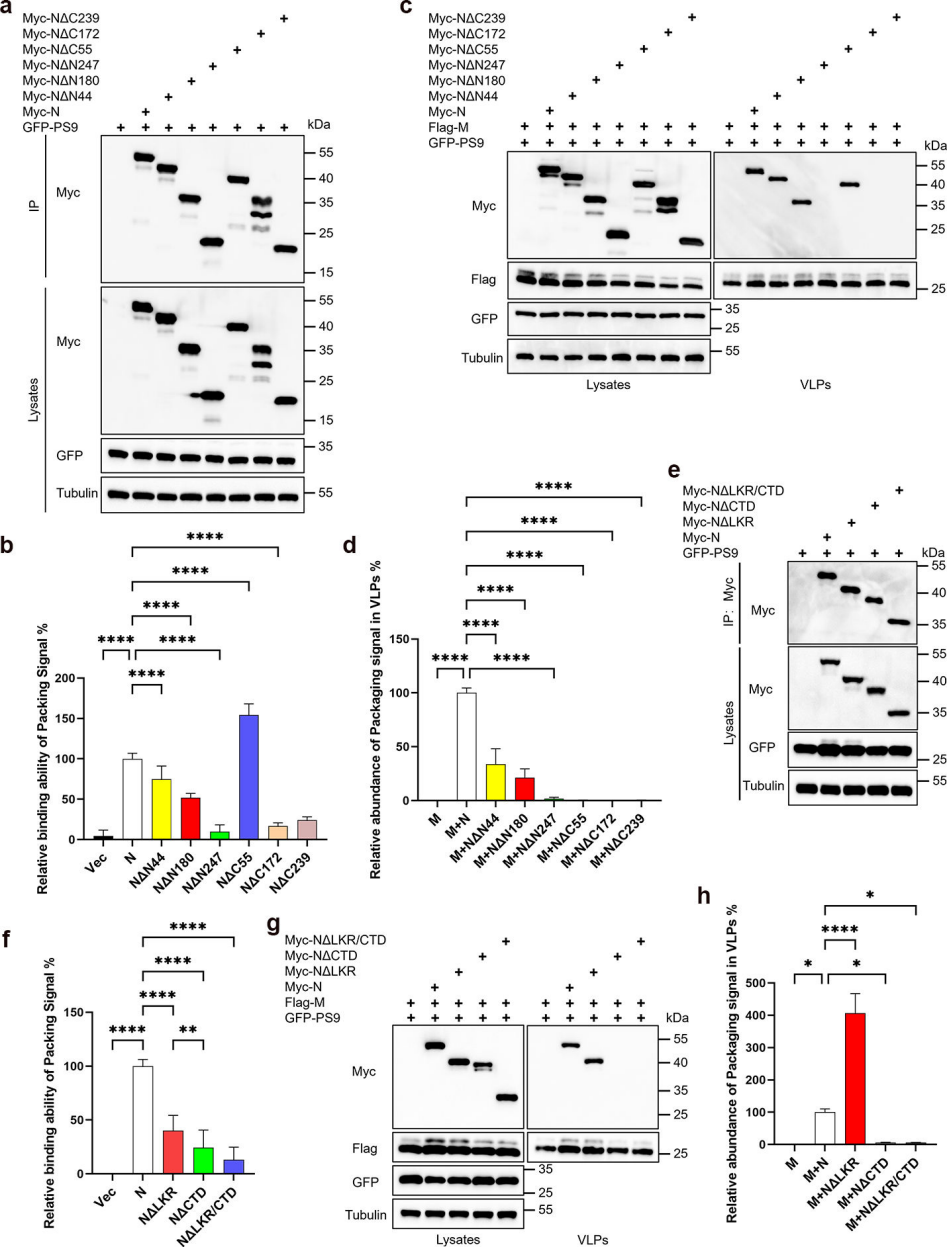
Multiple domains regulate N protein binding and packaging of genomic RNA. (a, b, e, f) Analysis of genomic RNA binding capabilities of N protein truncations. HEK293T cells were transfected with Myc-tagged N truncations and GFP-PS9 for 36 hours. Cell lysates underwent IP using anti-Myc antibodies, followed by RNA extraction and RT-qPCR. The data were analyzed on a relative scale, with the IP GFP RNA normalized against the cell lysate’s GFP RNA for each sample, b (*n* = 5), f (*n* = 4). IP and cell lysate fractions were further analyzed via WB. (c, d, g, h) Analysis of genomic RNA content in VLPs produced by N protein truncations and M protein. HEK293T cells were transfected with Myc-tagged N truncations and GFP-PS9, along with Flag-M, for 48 hours. Half of the cell culture supernatant was subjected to ultracentrifugation to isolate VLPs for WB analysis, while the remaining half underwent RNA extraction and RT-qPCR, d (*n* = 3), h (*n* = 5). Data in b, d, f, h were represented as mean ± SD and were analyzed by one-way ANOVA. *P < 0.05, **P < 0.01, ***P < 0.001, ****P < 0.0001.

Next, we examined the influence of various N protein domains on its binding affinity for genomic RNA. Our results showed that, compared to the full-length N protein, the binding ability of NΔN44, NΔN180, NΔN247, NΔC172, and NΔC239 to GFP-PS9 RNA is significantly reduced. Remarkably, deletion of the C-tail (NΔC55) significantly increased the N protein’s binding ability to GFP-PS9 RNA (Fig. 7a and b). Subsequently, we delved deeper into the mechanisms governing the N protein’s regulation of GFP-PS9 RNA encapsidation. Our observations revealed a notable decrease in GFP-PS9 RNA abundance in VLPs when ARM (NΔN44) was deleted individually or in conjunction with NTD (NΔN180) (Fig. 7c and d). NΔN247, NΔC172, and NΔC239 lacking budding ability and deficient in GFP-PS9 RNA binding, exhibited undetectable GFP-PS9 RNA in their VLPs. Intriguingly, GFP-PS9 RNA was absent in the VLPs of M+NΔC55 (Fig. 7c and d). We previously established that NΔC55 retains budding ability and displays stronger GFP-PS9 RNA binding than wild-type N. This suggests that the absence of the C-tail may impair the N protein’s ability to package GFP-PS9 RNA. Furthermore, the sole deletion of CTD alone significantly impaired the N protein’s binding ability to bind GFP-PS9 RNA, highlighting the critical role of CTD in the N protein’s interaction with GFP-PS9 RNA (Fig. 7e and f). Despite LKR deletion markedly reducing the N protein’s GFP-PS9 RNA binding ability, NΔLKR encapsulates GFP-PS9 RNA more effectively than the wild-type N protein (Fig. 7g and h). Conversely, NΔCTD completely lacks this capacity.

### N* exhibits an assembly and budding advantage

The emergence of N* (NΔN209) missing the N-terminal 209 amino acids has led to a reassessment of the SARS-CoV-2 assembly process. This variant is likely driven by R203K/G204R substitutions near the N start codon during the Alpha variant replication, which introduced a novel transcription regulatory sequence for NΔN209 transcription (40). Adly et al. demonstrated that assembly does not rely on the major RNA-binding sites of the NTD and the serine/arginine-rich region; instead, they identified the CTD as the primary RNA-binding site crucial for viral RNP assembly by NΔN209 (41).

**Fig 8:**
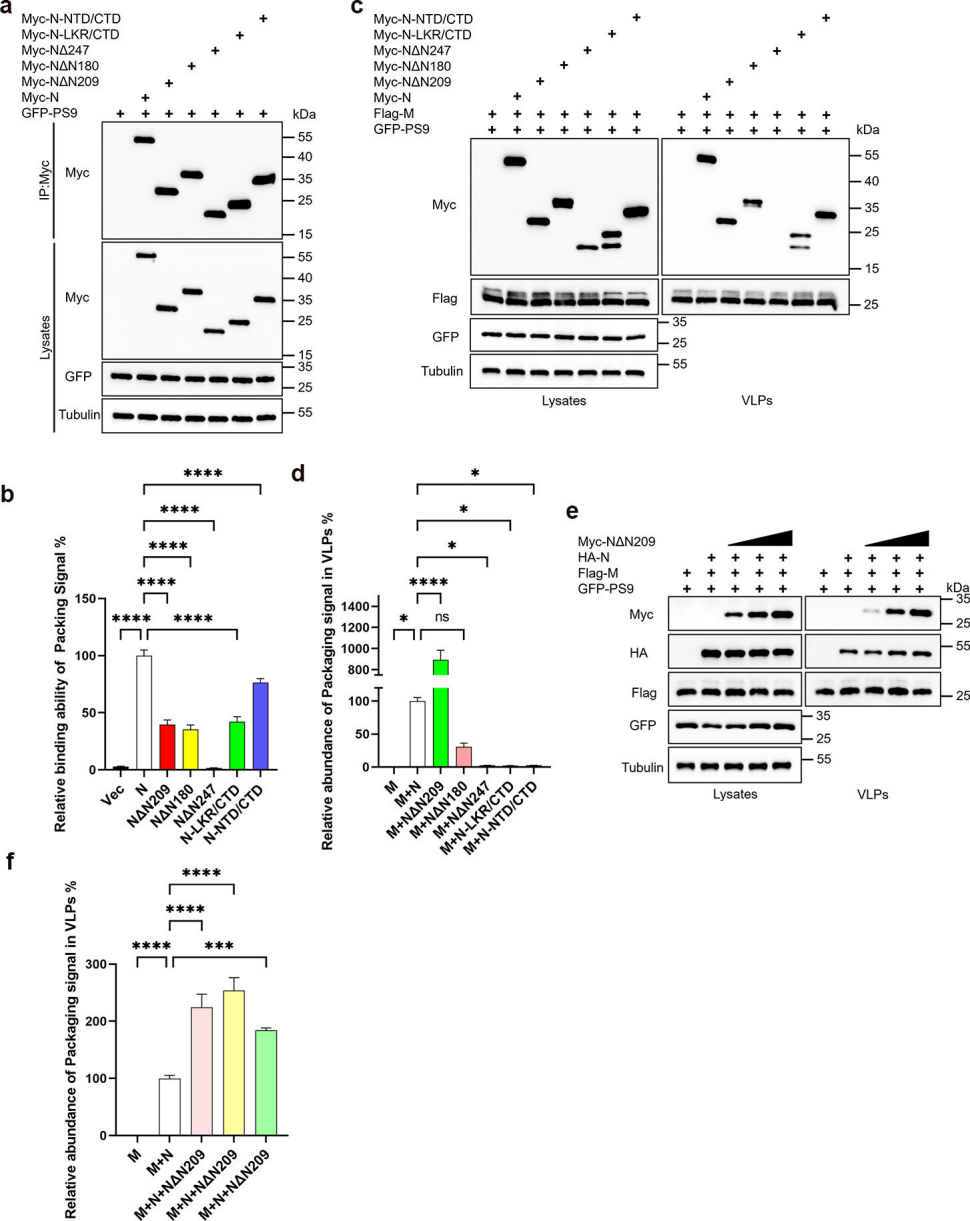
N* exhibits both the packaging of genomic RNA and budding capabilities. (a and b) Analysis of GFP-PS9 RNA binding capabilities of N protein truncations. HEK293T cells were transfected with Myc-tagged N truncations and GFP-PS9 for 36 hours. Cell lysates underwent IP using anti-Myc antibodies, followed by RNA extraction and RT-qPCR. The data were analyzed on a relative scale, with the IP GFP RNA normalized against the cell lysate’s GFP RNA for each sample, (*n* = 4). IP and cell lysate fractions were further analyzed via WB. (c and d) Analysis of GFP-PS9 RNA content in VLPs produced by N protein truncations and M protein. HEK293T cells were transfected with Myc-tagged N truncations and GFP-PS9, along with Flag-M, for 48 hours. Half of the cell culture supernatant was subjected to ultracentrifugation to isolate VLPs for WB analysis, while the remaining half underwent RNA extraction and RT-qPCR, (*n* = 5). (e and f) Impact of NΔN209 on GFP-PS9 RNA content within M+N-VLPs. HEK293T cells were transfected with increasing amounts of Myc-NΔN209, HA-N, Flag-M, and GFP-PS9 for 48 hours. Half of the cell culture supernatant was ultracentrifuged to isolate VLPs for WB analysis, while the other half underwent RNA extraction, followed by RT-qPCR, (*n* = 3). Data in b, d, and f were represented as mean ± SD and were analyzed by one-way ANOVA. *P < 0.05, **P < 0.01, ***P < 0.001, ****P < 0.0001.

Here, we also generated the NΔN209 mutant and conducted comprehensive validations. Interestingly, despite NΔN209 showing significantly weaker binding affinity to genomic RNA compared to the wild-type N protein (Fig. 8a and b), the relative genomic RNA content in its VLPs is nearly nine times higher than that in VLPs formed by the wild-type N protein and M protein (Fig. 8c and d). Additionally, we observed that both LKR/CTD and NTD/CTD exhibited enhanced binding affinity to genomic RNA compared to NΔN247 (Fig. 8a and b), indicating the involvement of NTD and LKR in N protein’s genomic RNA binding. However, lacking the C-tail, neither NTD/CTD nor LKR/CTD could efficiently encapsidate GFP-PS9 RNA (Fig. 8c and d). Furthermore, the introduction of NΔN209 significantly increases the GFP-PS9 RNA content in M+N-VLPs (Fig. 8e and f). The emergence of NΔN209 validates our conclusions and suggests a novel evolutionary mechanism for SARS-CoV-2, potentially enhancing genomic RNA packaging efficiency through N protein as an adaptive strategy.

### β-coronaviruses employ a similar assembly mechanism

**Fig 9:**
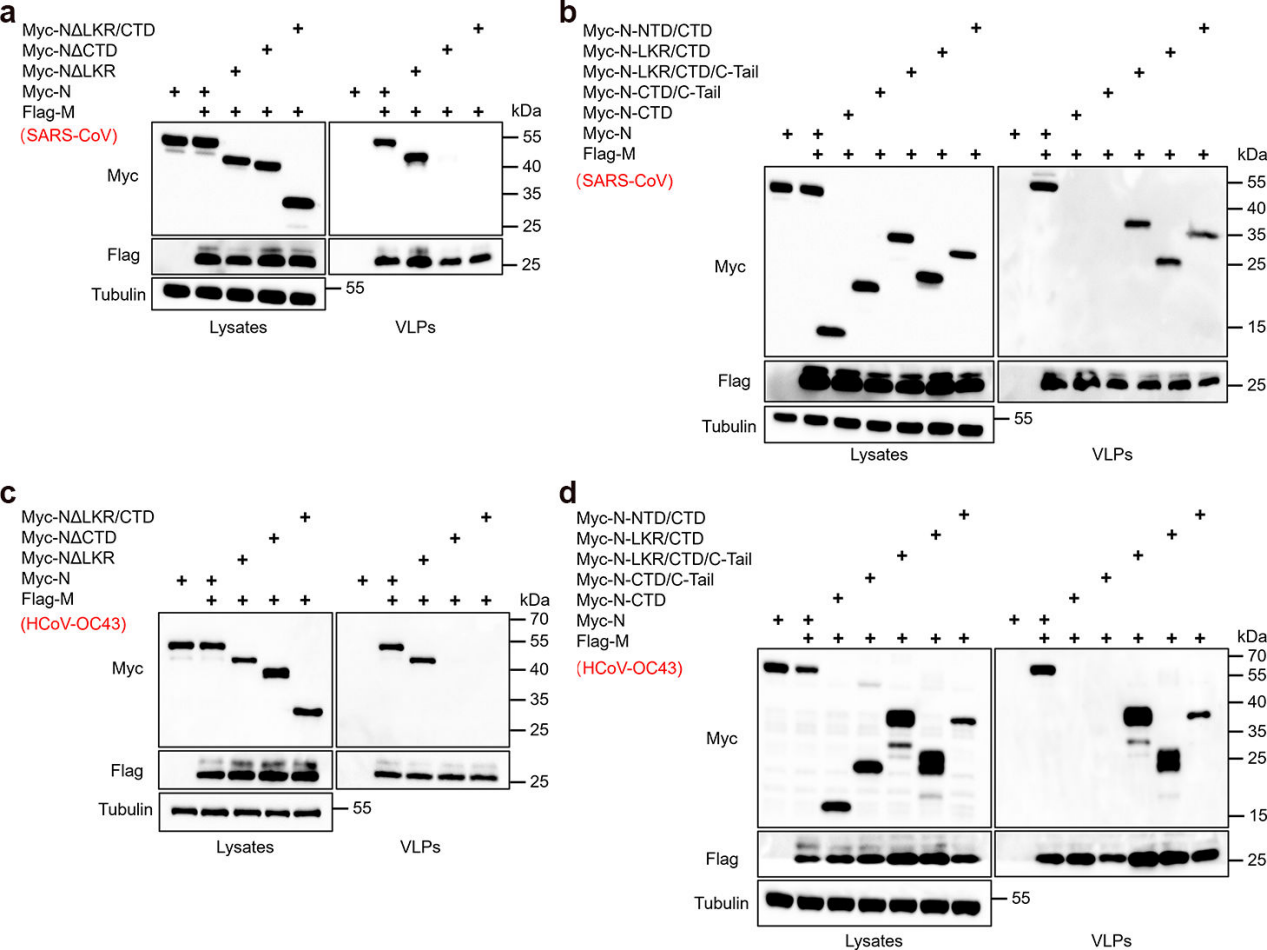
The assembly strategy of β-coronaviruses is comparable. (a–d) Analysis of the budding ability of N protein truncations of SARS-CoV and HCoV-OC43. HEK293T cells were transfected with Myc-tagged N truncations from SARS-CoV and HCoV-OC43, along with Flag-M for 48 hours. A VLPs budding assay was conducted using ultracentrifugation, followed by WB analysis.

The NTD and CTD of the β-coronavirus N protein are typically highly conserved, highlighting their crucial roles in the virus lifecycle, particularly in RNA binding and virus assembly (16, 44). Therefore, we aimed to investigate whether the assembly mechanism of the SARS-CoV-2 N protein, based on its CTD, is commonly found in β-coronavirus. Our findings reveal that the deletion of CTD alone in both SARS-CoV and HCoV-OC43 N proteins completely abolishes their budding ability (Fig. 9a and c). This suggests that in β-coronaviruses, the CTD of the N protein is indispensable for its budding assembly process. Moreover, similar to the results obtained for SARS-CoV-2, the CTD of N proteins from SARS-CoV and HCoV-OC43 viruses lacks the ability to bud when co-expressed with their respective M proteins. The assembly and budding capabilities are only restored after the fusion of CTD with its respective NTD or LKR (Fig. 9b and d). These results indicate that the assembly mechanism, based on the CTD and coordinated by multiple domains, is highly conserved across β-coronaviruses.

## DISCUSSION

**Fig 10:**
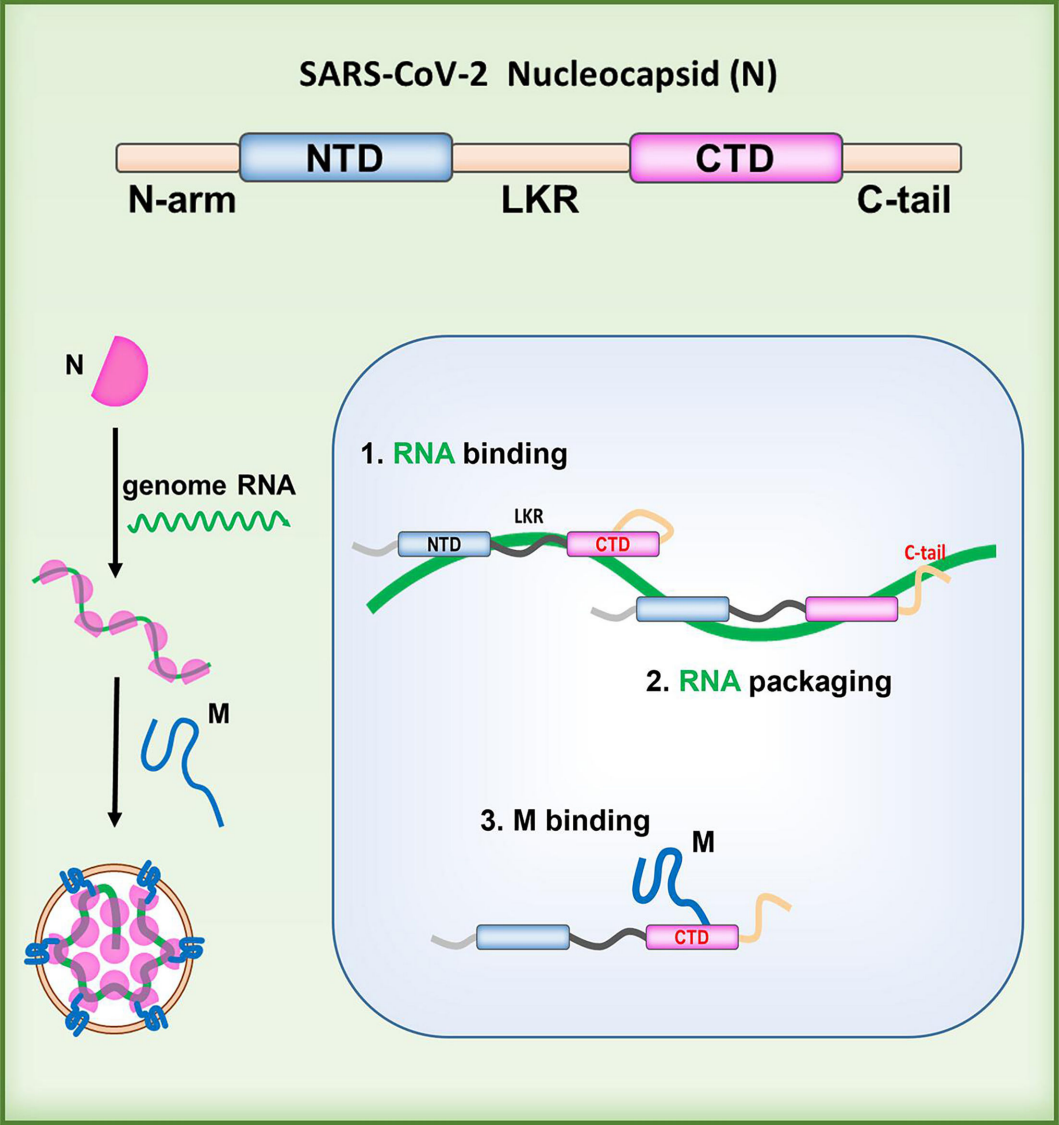
Diagram of the SARS-CoV-2 N protein-mediated viral assembly mechanism. The binding of genomic RNA is facilitated by the N protein’s CTD with the coordinated involvement of NTD and LKR. This is followed by the encapsidation of genomic RNA through the participation of the C-tail. Finally, the interaction between the CTD and the M protein, enabled by NTD and LKR, leads to the packaging of the RNP complex.

Mutations in the SARS-CoV-2 N protein play a critical role in the virus’s adaptive evolution, enhancing replication by modulating the assembly process involving the N protein. While previous research has extensively characterized the interactions between the N protein, M protein, and RNA—particularly focusing on the roles of the N protein’s NTD and CTD in RNA binding and the importance of N-M protein interactions in viral particle assembly—our study uncovers additional mechanisms underlying these processes. We found that the driving force for packaging the N protein into M-VLPs is the RNA-dependent interaction between the N protein’s CTD, conferred by the NTD or LKR, and the M protein. Additionally, efficient genomic RNA packaging relies on the assistance of the C-tail. These interactions collectively facilitate the packaging of the RNP (Fig. 10; Table 1). Notably, we found that the NTD of the N protein is not essential for assembly and genomic RNA packaging, introducing a degree of adaptability to the viral assembly process. These findings contribute to a more comprehensive understanding of the molecular mechanisms by which the N protein regulates viral assembly.

**TABLE 1:** Summary of results from N protein truncations^*a*^

WT/mutants	Budding (yes/no)	Oligomerization (yes/no)	M interaction (yes/no)	PS9-RNA binding	PS9-RNA packaging
N	Yes	Yes	Yes	Strong	Strong
NΔN44	Yes	Yes	Yes	Strong	Moderate
NΔN180	Yes	Yes	Yes	Moderate	Weak
NΔN247	No	Yes	Yes	None	None
NΔC55	Yes	Yes	Yes	Strong	None
NΔC172	No	No	No	Weak	None
NΔC239	No	No	No	Weak	None
NΔLKR	Yes	N/A	N/A	Moderate	Strong
NΔCTD	No	No	No	Weak	None
NΔLKR/CTD	No	N/A	N/A	None	None
NΔCTD1	Yes	Yes	N/A	N/A	N/A
NΔCTD2	No	No	N/A	N/A	N/A
NΔCTD3	No	No	N/A	N/A	N/A
NΔCTD2-1	Yes	Yes	N/A	N/A	N/A
NΔCTD2-2	No	No	N/A	N/A	N/A
NΔCTD3-1	No	No	N/A	N/A	N/A
NΔCTD3-2	Yes	Yes	N/A	N/A	N/A
NΔC299-339	No	No	N/A	N/A	N/A
NΔC301-307	Yes	Yes	N/A	N/A	N/A
NΔC308-315	Yes	Yes	N/A	N/A	N/A
NΔC316-328	No	No	N/A	N/A	N/A
NΔC329-339	No	No	N/A	N/A	N/A
N-CTD	No	Yes	No	N/A	N/A
N-LKR/CTD	Yes	Yes	Yes	Weak	None
N-NTD/CTD	Yes	Yes	Yes	Strong	None
N* (NΔN209)	Yes	N/A	N/A	Moderate	Strong
N (SARS-CoV)	Yes	N/A	N/A	N/A	N/A
NΔLKR (SARS-CoV）	Yes	N/A	N/A	N/A	N/A
NΔCTD (SARS-CoV）	No	N/A	N/A	N/A	N/A
NΔLKR/CTD (SARS-CoV）	No	N/A	N/A	N/A	N/A
N (HCoV-OC43)	Yes	N/A	N/A	N/A	N/A
NΔLKR (HCoV-OC43)	Yes	N/A	N/A	N/A	N/A
NΔCTD (HCoV-OC43)	No	N/A	N/A	N/A	N/A
NΔLKR/CTD (HCoV-OC43)	No	N/A	N/A	N/A	N/A

^*a*^ This table presents the effects of various truncations in the N protein on key functional aspects, including budding ability, oligomerization, interaction with the M protein, and genomic RNA (GFP-PS9 RNA) binding and packaging capabilities. WT N protein is used as a reference for comparison. The results are categorized as "Yes/No" for qualitative assessments (e.g., budding, oligomerization, M interaction). GFP-PS9 RNA binding/packaging is classified as strong (close to or above wild-type levels), moderate (40%–70% of wild-type levels), weak (10%–40% of wild-type levels), or none (indistinguishable from background). N/A indicates that the experiment was not performed for that specific truncation.

In line with previous studies (47), our findings confirm that the CTD of the N protein serves as the structural basis for its assembly and budding. Deletion of the CTD results in a marked reduction in the N protein’s oligomerization, RNA binding, and interaction with the M protein, underscoring its fundamental role in N protein function. Additionally, we found that removing the β1 and β2 regions within the CTD dimerization interface nearly abolishes the N protein’s oligomerization and budding capabilities (Fig. 3g through i), demonstrating a strong link between oligomerization and the protein’s ability to bud. However, the isolated CTD alone cannot interact with the M protein and initiate budding, suggesting that other domains regulate this process (Fig. 4a). The addition of the C-tail enables NΔN247 to interact with the M protein (Fig. 4c), consistent with previous findings where NΔN247 and the M protein’s CTD formed condensates (22). However, this interaction is insufficient to drive its packaging (Fig. 2b). We hypothesize that NΔN247, due to the absence of critical regions like the NTD or LKR, may lack the structural integrity and functional state necessary for successful packaging. While it can interact with the M protein, it fails to progress through the subsequent packaging steps. The involvement of NTD or LKR is crucial for facilitating the interaction between the CTD and the M protein, which is essential for successful packaging (Fig. 4f and g). These regions may help maintain the correct functional conformation of the N protein, providing both structural integrity and the necessary flexibility for effective engagement with the M protein. Our findings suggest that while the CTD alone, supported by the C-tail, can interact with the M protein, the absence of NTD or LKR prevents progression to successful packaging, implying their active role in driving the assembly process.

Previous research has shown that RNA can enhance the M-N interaction (24). Our study not only confirms this finding but also reveals that the interaction between NTD/CTD and LKR/CTD with the M protein is highly RNA-dependent. Compared to NΔN247, NTD/CTD, and LKR/CTD exhibit stronger RNA binding, suggesting that RNA may also play a role in maintaining the correct functional conformation of these mutants. We hypothesize that during viral infection, the coordinated action of the N protein’s NTD, LKR, and CTD enhances genomic RNA binding, thereby maintaining a conformation that is suitable for packaging by the M protein.

Multiple studies have shown that the N protein achieves genomic RNA packaging through phase separation, though these findings are often based on idealized binary (33–35). In a cellular environment with numerous non-specific RNAs, how does the N protein selectively bind and package genomic RNA? Our research confirms that the N protein exhibits strong specificity for genomic RNA binding and packaging into M+N-VLPs (Fig. 6). Further analysis revealed that, aside from the C-tail, all other domains on the N protein contribute to its binding to genomic RNA (Fig. 7a and b). Among these domains, the CTD serves as the foundation for N protein binding to genomic RNA (Fig. 7e and f), and the facilitation of genomic RNA binding by other domains depends on the presence of CTD. Interestingly, the C-tail negatively regulates RNA binding (Fig. 7a and b), possibly due to spatial hindrance, as seen in mouse hepatitis virus (MHV) studies (48). However, efficient RNA packaging also requires the C-tail (Fig. 7c and d). We believe this phenomenon may reveal the complex relationship between RNA binding and RNA packaging. Although RNA binding is a necessary prerequisite for RNA packaging, strong RNA binding alone does not necessarily ensure that RNA will be effectively packaged into VLPs. The C-tail may play a critical role in directing the bound RNA into the correct assembly pathway. The absence of the C-tail could impair the subsequent steps following RNA binding, preventing the N protein from effectively packaging RNA. Additionally, while the NΔC55 mutant can bind RNA and enter VLPs, it may lack the necessary structural or functional elements to stably encapsulate RNA within VLPs. To ensure that the N protein not only binds RNA but also guides it into the viral particle, the C-tail may play a crucial role in this process by regulating RNA folding, stabilization, or its interaction with the M protein (21). Further work is needed to elucidate the specific mechanisms by which the C-tail regulates the packaging of genomic RNA by the N protein.

Building on previous findings, it is clear that the NTD is not essential for N protein budding or genome packaging (Fig. 8a through d), as further supported by the NΔN209 mutant (41). However, the N-terminal region, including the N-arm, NTD, and the LKR, plays a role in genomic RNA binding, possibly contributing to viral genome replication and transcription (49). Deletion of this region may impair these functions, leading to reduced viral replication efficiency. This mutation likely represents a trade-off, streamlining the N protein to enhance assembly efficiency at the cost of replication capacity. In certain contexts, such as under immune pressure or where rapid viral particle assembly and release are critical, this trade-off may provide an adaptive advantage. Our study shows that NΔN209 retains strong genomic RNA packaging ability and increases genomic content within VLPs (Fig. 8c through f), suggesting that the benefits of efficient assembly and release may outweigh the drawbacks of reduced replication. Additionally, our research indicates that SARS-CoV and HCoV-OC43 employ an assembly mechanism similar to that of SARS-CoV-2 (Fig. 9).

In conclusion, this study reveals the coordinated roles of different N protein domains in genomic RNA binding, packaging, and interaction with the M protein during SARS-CoV-2 assembly. However, there are some limitations to this research. Firstly, we only used the N and M structural proteins to create VLPs, excluding S and E proteins as well as non-structural proteins like nsp3 that interact with the N protein (50). This limitation may lead to an incomplete understanding of the complex interactions involved in viral particle assembly. In future studies, we plan to incorporate these additional structural and non-structural proteins to build a more comprehensive SARS-CoV-2 assembly model, thereby validating our current findings and further exploring the complex mechanisms of viral assembly. Secondly, this study utilized constitutive promoters to drive the expression of N and M proteins, which may not fully reflect the natural expression levels during infection. To enhance the reliability of our results, we will consider using inducible expression systems or other methods in future studies to better mimic the conditions of natural viral infection.

## MATERIALS AND METHODS

### Cell culture

HEK293T and HeLa cells were acquired from the China Center for Type Culture Collection and were cultured in Dulbecco’s Modified Eagle Medium (DMEM, supplied by Gibco), which was enriched with 12% and 8% fetal bovine serum (FBS, also provided by Gibco), respectively. The cells were incubated at a temperature of 37°C in an atmosphere containing 5% CO_2_.

### Plasmid construction

The M and N proteins of both SARS-CoV and SARS-CoV-2 were acquired as plasmids from GenScript and underwent codon optimization. Subsequently, they were inserted into the pcDNA3.1(+) vector using PCR amplification and enzyme digestion techniques, accompanied by the addition of Flag and Myc tags at the N-terminus of the genes. On the other hand, the M and N proteins of HCoV-OC43 were procured through RNA extraction and reverse transcription from infected cells, followed by PCR amplification. Furthermore, all N gene mutants were created via PCR amplification utilizing N as a template and were integrated into the pcDNA3.1(+) vector, with a Myc tag appended at the N-terminus for identification. The deletion mutants of the N protein in SARS and HCoV-OC43 viruses were constructed based on homology analysis results described by Ya Peng et al. (18). The packaging signal of SARS-CoV-2 was amplified from plasmids containing the full genome of the virus, generously provided by professor Qiang Ding, and integrated into pcDNA3.1(+) along with a GFP carrying a stop codon at its N-terminus. Additionally, the sequence of random PS9 was generated by randomly shuffling the PS9 sequence using an online tool (NovoPro, Sequence information is provided in the supplementary materials). Random PS9 was synthesized by GenScript and inserted into pcDNA3.1(+) with mCherry fused at its N-terminus, which included a stop codon.

### SDS-PAGE and western blot

Cellular specimens were obtained and lysed using 500 µL of a specially formulated buffer containing 150 nmol/L NaCl, 50 nmol/L Tris-HCl (pH 7.4), 1% (vol/vol) Triton X-100, 1 mmol/L EDTA (pH 8.0), and 0.1% (wt/vol) sodium dodecyl sulfate (SDS), supplemented with a protease inhibitor cocktail. This mixture was then incubated under cryogenic conditions for 30 minutes. After incubation, centrifugation was carried out at 4°C, reaching a centrifugal force of 12,000 × *g* for 30 minutes to separate supernatants. The supernatants were then denatured by boiling in 5× SDS-PAGE loading buffer for 10 minutes. Subsequently, SDS-PAGE analysis was performed using a 10% resolving gel, followed by the electrotransfer of resolved proteins onto a nitrocellulose membrane obtained from GE Healthcare. The membrane was blocked using a 5% solution of skim milk powder in phosphate-buffered saline containing 0.1% Tween 20 (PBST) for 30 minutes. Following this, the membrane was incubated with a panel of primary antibodies, followed by exposure to horseradish peroxidase-conjugated goat anti-rabbit or anti-mouse IgG secondary antibodies sourced from Thermo Fisher Scientific for 1 hour. The primary antibodies were used at specified dilutions: mouse anti-Myc (1:5,000, CST, catalog #2276), mouse anti-Flag (1:5,000, CST, catalog #8146), rabbit anti-Flag (1:5,000, CST, catalog #14793), rabbit anti-Myc (1:5,000, CST, catalog #5605), rabbit anti-GFP (1:3,000, ABclonal, catalog AE011), rabbit anti-mCherry (1:3,000, ABclonal, catalog AE171), and mouse anti-Tubulin (1:5,000, ABclonal, catalog AC021). The secondary antibodies were used at a dilution of 1:5,000.

### Virus-like particle assays

HEK293T cell cultures were transfected with designated plasmids. After 48 hours post-transfection, cell culture supernatants were collected and clarified through centrifugation at 12,000 × *g* for 10 minutes. Subsequently, the supernatants were pelleted via ultracentrifugation through a 20% sucrose density gradient at 35,000 rpm, maintained at 4°C for 2 hours. Additionally, a portion of the supernatant was incubated overnight at 4°C with anti-Flag affinity gel (Sigma, catalog A4596) to capture the released VLPs. Cell lysates were prepared following established protocols for western blot analysis. The resulting VLP pellets were resuspended in PBS. Clarified supernatants, VLPs isolated using anti-Flag affinity gel, and cell lysates were denatured in 5× SDS-PAGE loading buffer by boiling for 10 minutes, followed by western blot analysis.

### Transmission electron microscopy

VLPs were obtained through the aforementioned immunoprecipitation method, followed by elution with a 3× Flag peptide solution (MCE, catalog HY-P0319). The eluate was then further concentrated using the previously described ultracentrifugation method. The final pellets were resuspended in PBS. VLP samples were absorbed onto a carbon-coated copper grid negatively stained with 1% phosphotungstic acid (pH 7.0) and then analyzed on a transmission electron microscope.

### Protease protection assay

VLP pellets were divided into four groups for treatment: untreated, 1% Triton X-100, 1 µg/mL trypsin, and a combination of Triton X-100 with trypsin. After incubation at 37°C for 30 minutes, the samples were denatured in 5× SDS-PAGE loading buffer for 10 minutes before proceeding to western blot analysis.

### *In vitro* co-immunoprecipitation

HEK293T cells were transfected using PEI (YEASEN, catalog 40816ES02) with designated plasmids. After 36 hours post-transfection, cell lysates were prepared in 500 µL of western blot buffer. In certain experiments, RNase A (Thermo Fisher Scientific, catalog EN0531) treatment was performed on cell lysates following the protocol provided in the instruction manual. A portion of 40 µL of lysates was kept as input, while the remaining lysates were incubated overnight at 4°C with either anti-Flag (Sigma) or anti-Myc (Biolegend) affinity gel under rotation. The beads were then pelleted at 5,000 rpm for 2 minutes at 4°C, washed three times in lysis buffer, and both the beads and input samples were boiled in 1× or 5× SDS-PAGE loading buffer, respectively, for 10 minutes in preparation for subsequent western blot analysis.

### *In vitro* RNA immunoprecipitation

HEK293T cells were cultured in 6 cm dishes and transfected with various amounts of plasmids, including 3 µg pcDNA3.1(+)-Myc-N, 3 µg pcDNA3.1(+)-mCherry-RPS9, and 3 µg pcDNA3.1(+)-GFP-PS9. To standardize DNA input, the pcDNA3.1(+) vector was added as needed. After 36 hours post-transfection, cells were lysed in 500 µL RNase-free lysis buffer and centrifuged at 12,000 × *g* for 30 minutes at 4°C. The resulting supernatants were divided into three parts: 8% for western blot analysis to assess plasmid expression, 12% for RNA analysis in 1 mL TRIzol, and the remaining portion for immunoprecipitation using anti-Myc antibodies. Following immunoprecipitation, the beads were washed thrice, resuspended in lysis buffer, and divided again: 6% for western blot analysis of immunoprecipitated proteins and the rest for RNA extraction using TRIzol. RNA samples, eluted in 20–30 µL RNase-free water, were quantified using a NanoDrop spectrophotometer to determine their concentrations.

### RNA quantification

Quantitative analysis of cellular and immunoprecipitated RNA was conducted using reverse transcription quantitative PCR (RT-qPCR). Initially, 500 ng of RNA was reverse transcribed into complementary DNA (cDNA) using a specific kit (Abclonal, catalog RK21400) following the provided protocol. Subsequent qPCR assays were employed to determine RNA levels, with results expressed on a normalized scale where both immunoprecipitated viral RNA (vRNA) and non-specific RNA quantities were adjusted relative to total cellular vRNA and non-specific RNA concentrations. qPCR primers specific to the GAPDH, GFP, and mCherry reporter genes were employed, specified as follows: GAPDH forward 5′-CCATGGGGAAGGTGAAGGTC-3′, reverse 5′-TGGAATTTGCCATGGGTGGA-3′; GFP forward 5′-AGATCCGCCACAACATCGAG-3′, reverse 5′-AACTCCAGCAGGACCATGTG-3′; mCherry forward 5′-CACGAGTTCGAGATCGAGGG-3′, reverse 5′-CAAGTAGTCGGGGATGTCGG-3′; Tubulin forward 5′-CGGGCAGTGTTTGTAGACTTGG-3′, reverse 5′-CTCCTTGCCAATGGTGTAGTGC-3′; Actin forward 5′-CACCATTGGCAATGAGCGGTTC-3′, reverse 5′-AGGTCTTTGCGGATGTCCACGT.

### Statistical analysis

Statistical analyses were performed using GraphPad Prism software (version 9.5). Student’s *t*-test was used to compare the means between the two groups. One-way analysis of variance (ANOVA) or two-way ANOVA was utilized to assess group differences, and the results are presented as mean ± SD. *P < 0.05, **P < 0.01, ***P < 0.001, ****P < 0.0001.

## Data Availability

All data generated or analyzed during this study are included in this published article and its supplemental material or are available from the corresponding author upon reasonable request.

## SUPPLEMENTAL MATERIAL

Supplemental material jvi.01036-24-s0001.docx Immunoprecipitation analysis of N protein and its mutants; randomly shuffled PS9 sequence.

ASM does not own the copyrights to Supplemental Material that may be linked to, or accessed through, an article. The authors have granted ASM a non-exclusive, world-wide license to publish the Supplemental Material files. Please contact the corresponding author directly for reuse.
