# Supplementary material for: SARS-CoV-2 N protein coordinates viral particle assembly through multiple domains
Source: J Virol. 2024 Oct 16;98(11):e01036-24. doi: 10.1128/jvi.01036-24 (PMC11575404; doi:10.1128/jvi.01036-24)
Supplement: Supplemental material — Immunoprecipitation analysis of N protein and its mutants; randomly shuffled PS9 sequence. [file jvi.01036-24-s0001.docx]

# Supplementary Materials for

**SARS-CoV-2 N protein coordinates viral particle assembly through multiple domains**

Yuewen Han，Haiwu Zhou，Cong Liu，Weiwei Wang，Yali Qin*，Mingzhou Chen*

Correspondence: Yali Qin. School of Life Sciences, Hubei University, Wuhan, China. Mingzhou chen. School of Life Sciences, Hubei University, Wuhan, China. Hubei Jiangxia Laboratory, Wuhan, China. Email: yqin@hubu.edu.cn & [chenmz@hubu.edu.cn](mailto:chenmz@hubu.edu.cn)

**This PDF file includes:**

Supplementary Fig. S1

Randomly shuffled PS9 sequence

**Fig S1. Immunoprecipitation Analysis of N Protein and Its Mutants.**


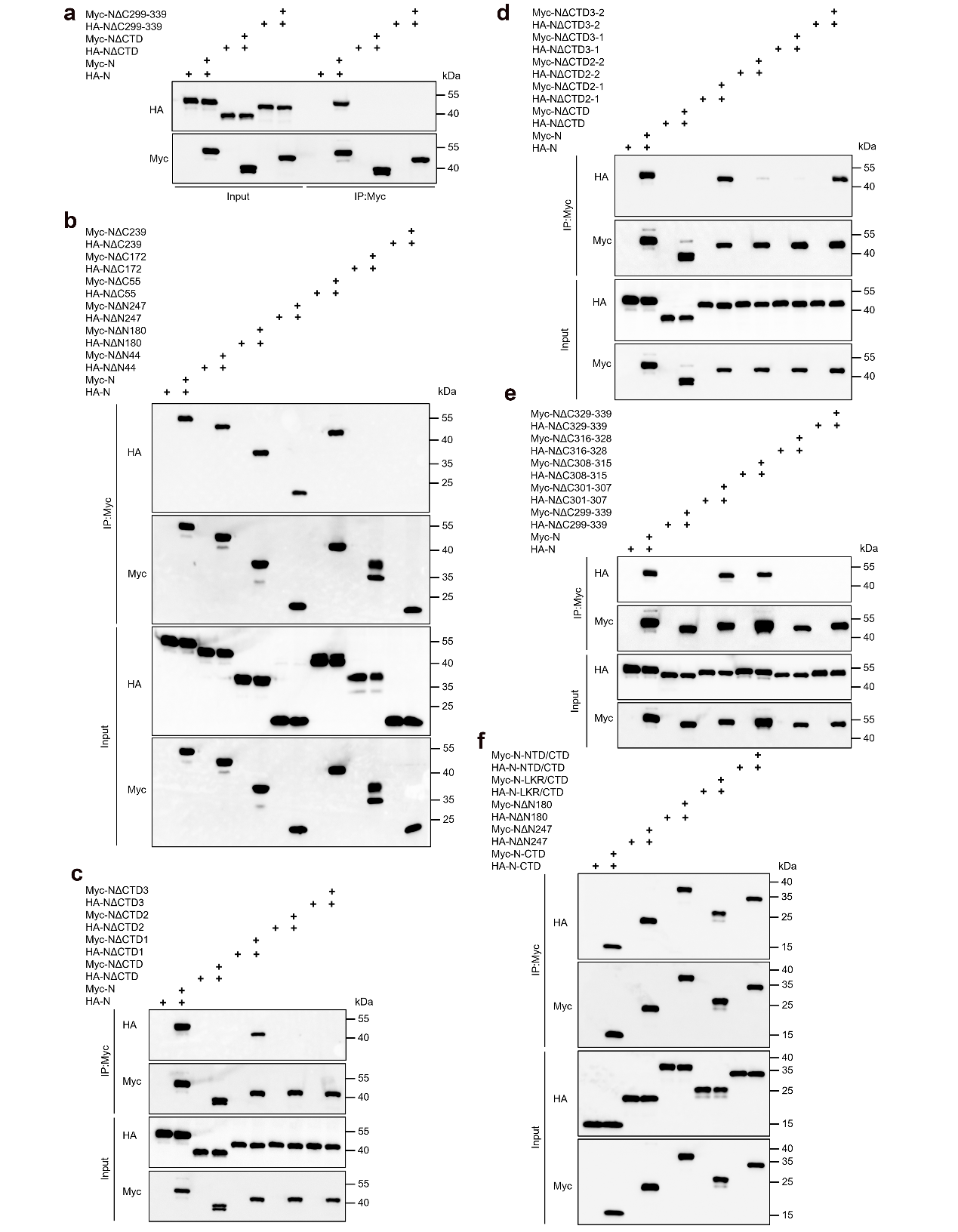


**Fig S1. Immunoprecipitation Analysis of N Protein and Its Mutants. a-f** HEK293T cells were transfected with HA-tagged N protein truncations and Myc-tagged N protein truncations for 36 hours, followed by Myc IP and subsequent WB analysis.

**Randomly shuffled PS9 sequence**：

>ATAAATAAGGAGCTATTGTATACACATAGAATGTCTAGGTTTTATAGGGTGCAACTGTGGTGTCTTATTCCGATTATCCAAACGGTGAAAAATACTAAGTTGCGGTAGTGAAATATTAGTTAACGTATTGATTCTTTTCTAATGCGTAAAATAAAAATGTTTCAAGAGTAGATGTTAAGAATGGAATTATATATTTTGCAATAATCAGCAGATATTTTAAAATACAGATGTGAGAAGGATCGTTCCTTTATAGCCTACTCTAAAACGTTACTTGTGTGTTCTTGATGCCTCAGATTCCGAACTAATTCAGTACATATTGGAGTTGCCCCACTCGGTCCACCAAAATTGTAAAGTACAGGATTTCTACTTACTCCACTACTACATTCACAAGTTGGGCATTGCGTTAGTTGAATCTTTGAGAGCAACATAGATTTATTATCTTGTACTATCAATGAGAATTATTATTGGACTATCAATAGAATGTGTAAACTAATAATCAAGTCCGGCGTTCGTTAAAAACCATCCAATCTTTACCTGAATATGATCGGCCTTTATGGATTCATAAATCTTGTGCTAGGACTATTACAATATACTGGTTGATTTTGATAATTCCTGTCCTAGTATCTTGATCAGTGTGTTGTTATTGGCTAATACATGTGACGGAGAGATAAAAACGAGATATATAGTAATCGGAAAGTAAGAGGGGTTAGGAAATCGAGATCGGCTGAAAATATTTTAATATAGGTGTTAATTCCGAACAAAGATCACGCACACTACTTATGGGAAATTATGAACTCGTCAAAAGTGTATCCTTAAATGGTCAATTAAGATAACGTAAATAGAGTTAAGTAGAGGACGTGTCAAGCAGCTATACTTCGAACATTTACCTGAGTGAAAACTCGCCCTAACTCAATTGGAGTTATTTTGAATGCTTATTCAGACTTTCATTACGCGAGACATATTGTTTCGCTCTAAATTGCATACTTAATCACAAACGTTTATAAGAATCATAGGCAGGGATGTTGATAGGAGTATTCAGTTTCGTGGTGTCAGATTACTGTTAGACATTTCAGTACAAGTATTGAGAAGCTA
